# Substance‐involved child‐to‐parent violence

**DOI:** 10.1111/dar.14011

**Published:** 2025-02-02

**Authors:** Travis Harries, Richelle Mayshak, David Skvarc, Christopher Eckhardt, Michelle Benstead, Peter Miller, Ashlee Curtis

**Affiliations:** ^1^ School of Psychology Deakin University Geelong Australia; ^2^ Department of Psychological Sciences Purdue University West Lafayette USA

**Keywords:** child development, family violence, substance use, parent–child relationship, parenting

## Abstract

**Introduction:**

Child‐to‐parent violence (CPV) is associated with youth substance problems; however, CPV which is *substance‐involved* (SU‐CPV) is specifically excluded from the consensus definition of CPV and few studies have explored the familial context surrounding SU‐CPV. This study utilised the I^3^ model to explore associations between parental monitoring, parental intrusiveness, affective reactivity, concurrent reactive CPV and SU‐CPV in an Australian community sample of caregivers.

**Methods:**

A total of 119 caregivers experiencing abusive CPV (frequent and severe) completed an online survey reporting on incidence of CPV from 12 to 24‐year‐olds under their care, parenting behaviours, traits of the young person and their experience of SU‐CPV.

**Results:**

Poor parental monitoring was positively associated with SU‐CPV, and this relationship was stronger at higher levels of affective reactivity in the young person. There were no significant three‐way interactions.

**Discussion and Conclusions:**

SU‐CPV may be most likely to occur where opportunity for child substance use is high, and risk of escalation is also high. These findings should be used to inform current CPV intervention, which may otherwise neglect youth substance use.

## 1 INTRODUCTION

Child‐to‐parent violence (CPV) is physical or non‐physical violence towards a parent or caregiver, by a young person under their care [1]. While the consensus definition of CPV technically excludes violent acts while the young person is influenced by substances [2], prior research suggests that the young person's substance use is often associated with CPV [3, 4]. As such, it is likely that a significant proportion of CPV is substance‐involved. Little is known about substance‐involved CPV (SU‐CPV) beyond basic prevalence; between 5–28% of justice/police involved CPV cases in Australia may involve the young person's use of alcohol, and 1–8% of cases involve their use of other substances [5, 6, 7]. While these prevalence estimates are small (though potentially under‐estimations), SU‐CPV may be common for those within CPV intervention, but rarely addressed [8]. For example, the majority of young person's entering the Break4change program (a parent–child group intervention for CPV) use substances at problematic levels [9].

The association between alcohol use and family violence has been documented across decades of research [10, 11, 12]. Collectively, this work suggests that the relationship between alcohol and violence perpetration is moderate in strength as well as highly nuanced, such that various risk factors and other intervening variables may moderate their association (for a review see Parrott and Eckhardt [13]). Threshold models outlining the aetiology of alcohol‐related family violence emphasise interactions among risk factors and assert that family violence occurs according to the relative balance of variables that ‘push’ for violence perpetration relative to those that ‘pull’ for its inhibition [12, 14, 15]. Alcohol use may impair processes that ‘pull’ for inhibition (e.g. emotion regulation) but only when interacting with other factors that promote a context for aggression (e.g. provocation; [16]) or ‘push’ for a specific violent response (e.g. high trait anger; [17]).

The I‐cubed (I^3^) model [16] is a prominent threshold model of substance‐related intimate partner violence [18] which posits that family violence is more likely when the strength of the urge to act aggressively exceeds the strength of the inhibitory forces counteracting this urge. According to the model, three key processes underlie the presence and/or degree of intimate partner violence perpetration: *i*nstigation, *i*mpellance and *i*nhibition [16]. Instigating factors are situational occurrences or events which present the opportunity for an aggressive response. Impelling factors are usually historical or temperamental factors which then increase the likelihood that the individual will *respond* to the instigating factor with violence or aggression. Inhibiting factors work against the influence of impelling factors to reduce the likelihood of aggression [16]. When instigating and impelling factors are strong but inhibition is weak, violence becomes more likely [16]. In the context of domestic violence (specifically alcohol‐related), the I^3^ model has been empirically supported across diverse samples, measurement and assessment techniques, and aggression paradigms [19, 20, 21].

Given that there is no direct literature on SU‐CPV to consult, the I^3^ model provides a framework to conject on the specific nature of SU‐CPV. Substance‐involved CPV requires the young person to be affected by prior or active substance use during the act of CPV. Given that young persons are unlikely to consume substances with their parents [22], we theorise that poor parental monitoring may provide the *opportunity* for substance use [23]. Specifically, parents who maintain less knowledge about, or awareness of, their child's activities or whereabouts may be less able to provide guidance around appropriate behaviour, but also provide the young person the autonomy and space to engage with substance use. When this is discovered, it may lead to arguments around unacceptable behaviours, eventuating in SU‐CPV [24]. Parents who are particularly intrusive (e.g. they may often go through the young person's belongings without their knowledge, among other intrusions of privacy; [25]) may be more likely to discover evidence of substance use; they may ignore developmentally appropriate boundaries and expectations of privacy. While high intrusiveness and low monitoring may appear to be (and often are) features of distinct parenting strategies, parenting in the context of CPV can often be inconsistent, characterised by switches between withdrawal (e.g. periods low monitoring) and confrontation (e.g. intrusiveness) [26]. Overall, the interaction between poor monitoring and intrusiveness may act as an indirect *instigator* of SU‐CPV via generation of interpersonal conflict.

Such conflict may be especially likely to escalate to SU‐CPV when the young person is particularly reactive or irritable (i.e. they demonstrate affective reactivity; [1, 27]). Additionally, affective reactivity may increase the likelihood of substance use [28], further perpetuating SU‐CPV. Dyads that more frequently experience reactive CPV are at risk because it may indicate that conflict has become an entrenched interaction pattern [29]. Reactive CPV occurs when the young person responds with hostility to perceived or actual hostility (including discipline) from the parent [26]. Over time, hostile interactions become a norm that the dyad can quickly re‐enter when disagreements or disciplinary actions arise (as per Granic and Patterson [29]). In this way, concurrent reactive CPV and affective reactivity may act as *impelling* factors.

Substance use is a commonly identified *(dis)inhibitory* factor in the I^3^ model [18], often attributed to its negative impact on executive function [30], and due to the crossover in brain structures and function implicated in substance use and aggressive behaviour [31]. For example, intoxication and chronic use of alcohol can hinder prefrontal and temporal functions including response inhibition, impulsivity, planning, use of environmental feedback, learning, working memory and goal selection [32, 33], and drug use (intoxication, chronic use and withdrawal) has been linked with impairments in attention, impulse control [34], planning [35] and in cognitive flexibility [36, 37]. Such impairments, coupled with the disruption in stress responses that can occur during withdrawal and abstinence [38], not only reduce the threshold for aggression and violence to occur, but may also act to increase the severity of such violence. Thus, an act of SU‐CPV is inherently disinhibited and, indeed, several qualitative CPV studies have reported that withdrawal, abstinence and intoxication contribute to SU‐CPV [27, 39, 40].

An explanatory model of SU‐CPV has not yet been empirically explored in the literature. The combination of poor parental monitoring and intrusiveness (high instigation), concurrent reactive CPV and/or affective reactivity (high impellance), alongside the cognitive effects of substances (low inhibition) may increase the likelihood of SU‐CPV. The aim of this study was to explore these associations. Two initial hypotheses were tested: (i) we hypothesised that SU‐CPV would be positively associated with poor parental monitoring; (ii) we hypothesised that this positive association to be strengthened by higher levels of parental intrusiveness, affective reactivity in the young person and concurrent reactive CPV in the dyad. We also tested the perfect storm hypothesis directly with a third hypothesis, by exploring three‐way interactions; (iii) we hypothesised that there would be a significant three‐way interaction between poor monitoring, intrusiveness and affective reactivity/reactive CPV in their relationship with SU‐CPV, whereby higher levels of all three variables would produce the strongest relationship. We note that in all models, inhibition was inherent to the dependent variable (SU‐CPV).

## 2 METHOD

### 2.1 Participants

The sample comprised 119 caregivers, aged between 27–78 years (*M* = 48.4, SD = 7.34; 97% female) and reporting on young people aged 12–24 years (*M* = 15.94, SD = 3.25; 40% female). Participants were recruited through online advertisements on social media for a study on ‘parent–child conflict’. Most participants were a biological parent of the young person (87%). Others were a step, foster, or adoptive parent (5.9%), or a legal guardian (0.8%). All participants in the sample were experiencing abuse‐level CPV (see Section 2.2 [ABC‐I] for criteria); those who were not experiencing substantial and ongoing CPV were excluded prior to formation of the sample, as were those with young persons aged less than 12 years. While this study is not directly related to intervention, the high severity of CPV experienced in our sample means that they represent families who are most likely to receive referral to formal intervention, if they were to seek help.

### 2.2 Materials

#### 2.2.1 Child‐to‐parent violence

The *form* of CPV was measured using the Abusive Behaviour by Children—Indices (ABC‐I) which assesses verbal, coercive and physical CPV [41]. The ABC‐I distinguishes between abusive and non‐abusive CPV, with a cut‐off score of 16. As per the recommended scoring method, behaviours are weighted differently depending on their severity to distinguish abuse from violence; higher frequency for low severity behaviours is required to meet the cut‐off. For example, verbal aggressions (considered in isolation; e.g. ‘shouted or swore’) needed to be reported daily to be defined as abuse, whereas any report of physical aggression (e.g. ‘pushed, grabbed, punched …’ etc.) in the past 12 months constituted abuse. Reliability estimates were not calculated as the ABC‐I is an index.

The *function* of CPV was assessed using the CPV‐F [1], which captures reactive (*α* = 0.82), proactive (*α* = 0.94) and affective (*α* = 0.62) CPV across 18‐items measured on a 5‐point scale (0 = never, 4 = almost always). Proactive CPV is planned and instrumental (e.g. ‘The young person tries to intimidate me (e.g. threaten violence) to get their way’); reactive CPV is retaliatory against perceived or actual threat (from the parent; e.g. ‘The young person reacts violently or aggressively when I punish them for something they have done wrong’); and affective CPV is a behavioural response displaced from internal fear or frustration (e.g. ‘When the young person is being violent or aggressive towards me … they appear anxious, frightened, or scared’) [26]. For theoretical reasons, we only used the reactive CPV subscale in inferential models (this function is most relevant as an impelling factor in the I^3^ model).

A novel scale was developed to measure SU‐CPV, given that there was no pre‐existing measure. We conducted a rapid review of qualitative CPV literature [27, 40, 42, 43, 44, 45] to identify how the young person's substance use may be implicated in incidents of CPV. We developed three items to reflect these themes: ‘When the young person is being violent or aggression toward me …’ 1. ‘… they are *coming down* from drug or alcohol use’, 2. ‘… they had been *withholding or abstaining* from their usual drug or alcohol use’ and 3. ‘… they are *under the influence* of drugs or alcohol’. The substance‐involved nature of CPV was measured using three items on a 5‐point scale (0 = never, 4 = almost always). A latent variable was formed by calculating the average response to these items; there were strong inter‐item correlations (*r* = 0.68–0.96), and reliability was excellent (*α* = 0.92).

#### 2.2.2 Parent factors

Parental monitoring was measured using the Alabama Parenting Questionnaire [46]. The Alabama Parenting Questionnaire is a measure of parenting behaviours consisting of 42 items across five dimensions of parenting, measured on a 5‐point Likert scale ranging from ‘never’ to ‘almost always’. Only the monitoring/supervision subscale (six items; e.g. ‘Your young person goes out without a set time to be home’) was utilised (*α* = 0.86).

Parental intrusiveness was measured using the Parental Boundaries Scale [25], which is a 35‐item parent‐report scale designed to measure boundary dissolution within the parent–child relationship. The intrusiveness subscale was used (e.g. ‘I look through my young person's personal things without his/her knowledge’), with items measured on a 5‐point Likert scale ranging from ‘never’ to ‘almost always’. Reliability for the intrusiveness subscale was low but acceptable (*α* = 0.58).

#### 2.2.3 Child factors

Affective reactivity (i.e. anger) was measured using the Affective Reactivity Index [47] consisting of seven items (e.g. ‘overall irritability causes him/her problems’) measured on a 3‐point scale ranging from ‘not true’ to ‘certainly true’. Reliability was excellent in the current study, *α* = 0.93.

### 2.3 Procedure

Ethical approval was attained from a university ethics board (ID: 2020‐227). Participants completed an online survey hosted on Qualtrics lasting approximately 25 min. Participants provided implied informed consent via their commencement of the survey, after being shown the Plain Language Statement. Data were analysed using IBM SPSS version 28 [48].

#### 2.3.1 Analysis

A priori power analyses suggested that a sample of at least 114 participants was required to detect a moderate effect (*F* ^2^ = 0.10) at 80% power, with an alpha of 0.05. Materials and analysis code for this study are not available due to ethical restrictions. We built a negative binomial model to explore bivariate relationships and two‐ and three‐way interactions between parental monitoring and intrusiveness, reactive CPV, and affective reactivity, controlling for the age and gender of the young person. Negative binomial models allow modelling of highly variable but uncommonly occurring outcome variables (such as SU‐CPV) without sacrificing statistical power [49].

## 3 RESULTS

The mean, standard deviation or frequencies and percentages for key variables used in the inferential models are presented in Table 1. SU‐CPV was relatively uncommon in frequency when compared to other functions of CPV. However, 25% of participants reported experiencing some degree of SU‐CPV. This is in line with the upper estimate of justice/police‐reported prevalence estimates [6, 7, 50]. SU‐CPV was most strongly correlated with poor parental monitoring, affective reactivity and reactive CPV. The age of the young person was positively correlated with SU‐CPV and poor monitoring, but negatively correlated with frequency of CPV in general; it may be that CPV reduces as the young person gets older but the likelihood of this becoming substance‐involved increases. All correlations are presented in Table 1.

**TABLE 1:** Correlations between key variables and descriptive statistics.

	*M*	SD	1. Gender	2	3	4	5	6	7	8	9	10	11	12
2. Age	15.94	3.25	−0.06											
3. SU‐CPV	0.47	0.92	0.09	0.41**										
4. Poor monitoring	7.63	5.79	0.14	0.59**	0.68**									
5. Affective reactivity	7.93	4.29	0.08	−0.08	0.24*	0.24*								
6. Intrusiveness	8.85	3.02	0.09	−0.10	0.09	0.04	0.09							
7. Reactive CPV	1.82	0.86	0.11	−0.05	0.27**	0.26*	0.65**	0.21*						
8. Proactive CPV	1.58	0.94	0.24**	−0.03	0.25**	0.25*	0.63**	0.15	0.77**					
9. Affective CPV	2.19	0.85	0.05	0.07	0.08	0.03	0.18	0.20*	0.11	0.03				
10. CPV total	183	188	0.11	−0.19*	0.15	0.15	0.50**	0.05	0.64**	0.66**	0.10			
11. CPV verbal	14.14	9.95	0.02	−0.17	0.07	0.08	0.50**	0.12	0.60**	0.58**	0.11	0.74**		
12. CPV coercive	95.83	89.47	0.08	−0.16	0.18	0.15	0.55**	0.04	0.66**	0.70**	0.12	0.96**	0.77**	
13. CPV physical	72.87	98.62	0.13	−0.20*	0.11	0.14	0.41**	0.04	0.56**	0.62**	0.07	0.96**	0.62**	0.85**

*Note*: Mean (*M*), standard deviation (SD), age and gender refer to the young person, Reference category for gender is male.

Abbreviations: CPV, child‐to‐parent violence; SU‐CPV, substance‐involved child‐to‐parent violence.

* *p* < 0.05;

** *p* < 0.01.

### 3.1 Inferential statistics

The results of the negative binomial model are presented in Table 2. While age was initially correlated with SU‐CPV, any effect of age was accounted for by its correlation with poor monitoring. Poor monitoring (odds ratio 1.38, *p* < 0.001) held the only significant main effect. Regarding interactions, the relationship between monitoring and SU‐CPV was significantly moderated by affective reactivity (odds ratio 1.03, *p* = 0.010), whereby the relationship between monitoring and SU‐CPV was stronger at higher levels of affective reactivity (see Table 3). There were no significant three‐way interactions. To make sense of this, we explored patterns within the simple effects and found that intrusiveness may not be contributing unique effects to the outcome; three‐way interaction was only present where the young person was also high on affective reactivity.

**TABLE 2:** Negative binomial regression model predicting substance‐involved child‐to‐parent violence.

	*β*	SE	Exp (*B*)	*p*‐value
Gender	−0.01	−0.18	1.39	0.533
Age	0.04	0.40	0.99	0.818
Poor monitoring (Mon.)	**0.53**	**5.16**	**1.38**	**<0.001**
Intrusiveness (Int.)	−0.01	−0.07	0.95	0.913
Reactive CPV (R‐CPV)	0.22	1.99	2.90	0.058
Affective reactivity (AR)	0.08	0.77	0.85	0.787
Mon*Int	−0.01	0.02	0.99	0.658
Mon*R‐CPV	−0.13	0.07	0.88	0.061
Mon*AR	**0.03**	**0.01**	**1.03**	**0.012**
Mon*Int*R‐CPV	0.01	0.02	1.01	0.710
Mon*Int*AR	<0.01	0.01	1.00	0.763

*Note*: Reference category for gender is male. Bolded lines are statistically significant.

Abbreviation: CPV, child‐to‐parent violence.

**TABLE 3:** Conditional effects of monitoring on substance‐involved child‐to‐parent violence at different levels of affective reactivity.

Affective reactivity	*β*	SE	Exp (*B*)	*p* (confidence interval)
−1·SD	0.21	0.06	1.228	<0.001 (1.09, 1.38)
Mean	0.32	0.05	1.38	<0.001 (1.24, 1.53)
+1·SD	0.44	0.08	1.55	<0.001 (1.32, 1.82)

## 4 DISCUSSION

We aimed to explore correlates of SU‐CPV using the I^3^ framework, broadly predicting that, in families experiencing abusive CPV, SU‐CPV would be more likely to the extent that instigating (poor parental monitoring and intrusiveness) and impelling factors (concurrent reactive CPV and/or affective reactivity) were strong, with inhibition in our model being inherent to substance use. We found that poor parental monitoring was positively and strongly associated with SU‐CPV, supporting the first hypothesis. We further hypothesised that this relationship would be moderated by instigating (intrusiveness) and impelling factors (affective reactivity and Reactive CPV). In partial support of this, we found that the relationship between monitoring and SU‐CPV increased in strength where there were higher levels of affective reactivity in the young person (high impellance). There were no other significant interactions.

Greater autonomy afforded through poor parental monitoring may create opportunities for substance use and SU‐CPV, and where the young person also has high affective reactivity, the likelihood of SU‐CPV is further increased. This aligns with prior literature which finds that parenting styles with lower involvement attract greater risk of CPV compared to those where boundaries are appropriately enforced [42, 51]. The young person's affective reactivity may be exacerbated by substance use and increase the risk of SU‐CPV; intense irritability or anger can inhibit an individual's ability to override aggressive responses while effected by alcohol [13]. Indeed, the impact of alcohol on aggression may be realised through higher levels of trait anger [52]. Thus, our findings align with general aggression literature [13] but also need to be considered within the context of the population sampled, whereby all families were experiencing abusive CPV. Young people who use CPV and experience substantial frustration and irritation could be coping by using substances [53]. CPV generates ongoing stress and conflict as well as shame and guilt for those involved [54, 55]. These experiences increase the risk of substance use, substance craving and further hostility, potentially leading to a negative feedback loop may form, perpetuating further shame and guilt, substance use and CPV [56, 57].

We theorised that poor monitoring (i.e. opportunity) may interact with intrusiveness to provide instigation. We did not find support for this prediction and as such, instigation is not clear from our models. Intrusiveness was inconsequential when considering the impellance of affective reactivity; instigation may be inherent to, or highly concomitant with, affective reactivity. For example, affective reactivity is associated with hostile attribution bias that may attract conflict to very minor or misperceived transgressions [58]. The myopic effects of alcohol or other substances may exaggerate this bias [59]. On the other hand, it may be that there are methods of instigation we have not conceptualised in our models, such as tactics of discipline that may be more likely perceived as provocation. For example, power‐assertive discipline strategies are a demonstrated correlate of CPV [60].

CPV is multifunctional; it is motivated within a variety of situations and intents [26]. It may be that the young person is also initiating conflict involving SU‐CPV (i.e. it is proactive). For example, this could be via requests for money or substances, or other unreasonable demands while affected by substances, particularly when withdrawing or enduring abstinence, circumventing the necessity of parental intrusion to generate conflict. In this study, SU‐CPV shared a positive and significant correlation with CPV that is motivated by a proactive function, as well as a reactive function.

### 4.1 Limitations and future directions

Our sample was small, so it may be that some smaller effects were not detected. However, the sample was responsibly restricted to ensure translation of findings to those most likely to benefit from them, which resulted in the smaller, but more specific sample; all our participants were experiencing CPV at an abuse‐level, not once‐off minor incidents. Thus, the sample represents those most likely to warrant formal intervention. Any variables with weaker effects than those detected are likely not efficient intervention targets. Nonetheless, future enquiries into SU‐CPV, and CPV, in general, should work to attain larger samples, without over‐representation of control or minor CPV cases.

The current study relied on caregiver report of parenting behaviour which can be susceptible to biases (e.g. social desirability). Observational methods of assessment may provide a more objective perspective, though the feasibility for this methodology for observing CPV must first be established. The current study was also cross‐sectional; we cannot confirm that the process of poor parental monitoring creating opportunity for substance use, and affective reactivity leading to SU‐CPV following substance use, is indicative of a causal relationship. For example, it is reasonable to speculate that some parents experiencing CPV may withdraw monitoring over time to protect themselves and other family members (e.g. siblings). A longitudinal study, diary approach or ecological momentary assessment is necessary (e.g. [61]) to explore directionality. Alternatively, the critical incident technique (e.g. [62]) could be used to understand the processes leading up to SU‐CPV. For example, this direction could help to understand how parent responses to substance use or intoxication, or to SU‐CPV itself, may feed into the conflict process (e.g. the use of corporal punishments; [63]). When doing so, it is important to understand the nature of the young person's substance use (i.e. what and where) and ensure this fits appropriately within our conceptualisation; we could not measure this due to the restrictions associated with caregiver‐report.

## 5 CONCLUSION AND CLINICAL IMPLICATIONS

In families experiencing CPV, incidents which are substance‐involved may be most likely to occur where opportunity for substance use is high, and risk of conflict escalation is high too (i.e. higher affective reactivity). Our findings generally align with prior theories for SU‐CPV and general aggression literature. Nonetheless, this is the first inferential study of SU‐CPV and provides precedent and initial direction for future enquiry, as well as potential intervention targets for current CPV intervention, where substance use is identified. Working to support parental monitoring of the young person's behaviour could also reduce the opportunities for substance use, and the conflict which may arise from this. Adjunctly, working to reduce impellance may lessen the impact of poor monitoring on SU‐CPV, by reducing the likelihood that conflict may escalate to violence. Affective reactivity in the young person can be reduced through both pharmacological and non‐pharmacological (e.g. modified cognitive behavioural therapy; [64]) treatments [65].

Reduced parental monitoring may be a consequence of ongoing conflict and parental helplessness developed therefrom. Attempting to increase parental monitoring may reduce the opportunities for substance use, but may also precipitate further CPV, albeit not directly substance‐involved (though perhaps CPV arising from restricted access to substances could also constitute SU‐CPV; [27]). This is particularly likely when the young person demonstrates irritability, which they could be using substances to relieve. Given this, there may be a strong intervention need to consider SU‐CPV alongside other functions of CPV. For example, in addressing concurrent reactive CPV, SU‐CPV may also be reduced by improving the flexibility of dyads to avoid interactions which include aggression.

Targeting substance use directly may be important, including the reasons for substance use. For example, the young person may be using substances to cope with an unpredictable and harsh family environment; the majority of young people who use CPV have experiences of trauma [66]. We found that SU‐CPV was prevalent in this sample of families experiencing abuse‐level CPV, who may benefit from general CPV intervention programs with adjunct substance use components. Indeed, substance use is found to be a barrier to treatment success within some intervention programs that do not directly address this co‐morbidity (e.g. the KIND program; [5]). Substance use should be approached as a potential contributor to incidents of CPV within intervention, rather than a separate issue.

## AUTHOR CONTRIBUTIONS

All authors certifies that their contribution to this work meets the standards of the International Committee of Medical Journal Editors.

## FUNDING INFORMATION

This study was funded by the School of Psychology, Deakin University.

## CONFLICT OF INTEREST STATEMENT

The authors have no conflicts of interest.

## Data Availability

Data available on request due to privacy/ethical restrictions.
